# Serum periostin levels in early in pregnancy are significantly altered in women with miscarriage

**DOI:** 10.1186/s12958-017-0307-9

**Published:** 2017-11-02

**Authors:** A. Freis, J. Schlegel, R. J. Kuon, A. Doster, J. Jauckus, T. Strowitzki, A. Germeyer

**Affiliations:** Department of Gynecological Endocrinology and Fertility Disorders, University Hospital Heidelberg, INF 440, 69120 Heidelberg, Germany

**Keywords:** Early pregnancy, Miscarriage, Biomarker, Periostin

## Abstract

**Background:**

Miscarriage is a common complication in pregnancy and there is still a lack of biomarkers usable in asymptomatic patients before the event occurs. Periostin (PER), whose levels rise particularly during injury or inflammation, has been shown to play an important local role in implantation and early embryonic development. As PER has been described as a biomarker in various medical conditions we intended to evaluate if changes in PER serum levels may help to identify women at risk for spontaneous abortion in the first trimester.

**Methods:**

Women between 18 and 42 years without confounding comorbidities who conceived by IVF/ICSI and ovarian hyperstimulation were analysed in the study after informed consent. Maternal serum samples from 41 patients were assessed at the time of pregnancy testing (PT) and the following first ultrasound checkup (US). Patients were subsequently divided in two groups: (1) patients with subsequent miscarriage in the first trimester (*n* = 18) and (2) patients with ongoing pregnancy (*n* = 23), allowing for statistical analysis and investigating the change of PER levels per individual. PER levels were measured using enzyme-linked immunosorbent assay. Statistical analysis was performed using the Fisher exact and Student’s t test. *p* ≤ 0.05 was considered to be significant.

**Results:**

There was no significant difference concerning possible confounders between the two groups. We did not find any significant difference in PER levels at the time point of PT or US. By investigating the interindividual changes of PER between the two time points however, we observed that patients with a following miscarriage showed increasing levels of PER at the time point of PT compared to US in contrast to patients with an ongoing pregnancy who demonstrated a decrease in PER levels. These alterations were significant in the absolute as well as in the relative comparison.

**Conclusion:**

The relative expression of PER between PT and US is significantly altered in asymptomatic women with subsequent miscarriage compared to women with ongoing pregnancy. Therefore systemic PER levels might represent a potential promising biomarker for the assessment of pregnancy outcome.

**Trial registration:**

Not applicable.

## Background

Despite the lack of consistent definitions, miscarriage (spontaneous abortion) is often defined as intrauterine pregnancy demise confirmed by ultrasound before the 24th week of gestation [1]. Miscarriage is a common complication in pregnancy and affects about 8 to 20% of clinically recognized pregnancies in the first 20 weeks of gestation [2]. Among others, the causes of early pregnancy loss are various, including cytogenetic abnormalities [3] as well as placental development, advanced maternal age [4], previous miscarriage [5], body mass index before pregnancy less than 18.5 or above 25 kg/m2 [6] as well as heavy maternal smoking [7].

As the fetus represents a semi-allograft, immune mechanisms are discussed as contributing factors.

Periostin (PER) is a 90 kDa extracellular protein which is expressed in multiple compartments of the body, especially in aorta, stomach, lower gastrointestinal tract, placenta and uterus [8]. Recent studies have been published evaluating its role in skin, bone, kidney, heart, lung, blood vessel, and allergic reactions, as well as cancer [8–10]. Levels rise particularly during injury or inflammation reactions. Furthermore, PER is a ligand for ανβ3- and ανβ 5-integrins, proteins relevant in implantation [11], and promotes cell motility [12, 13]. Its expression is triggered by transforming growth factor (TGF)-b, IL-4, and IL-13 [14, 15].

Of note many mechanisms of embryo implantation have first been described in cancer and metastasis. In oncology PER plays a crucial role especially in metastasis where it has been shown to increase Wnt signaling, a pathway that is also involved in embryo implantation [16] and in cancer stem cells [17, 18].

PER seems to be essential for metastatic cell maintenance and blocking PER results in metastasis prevention [19]. Another possible pathway of PER in facilitating metastasis is the formation of the immunosuppressive premetastatic niche formation [20].

In order to evaluate the regulation of PER during early pregnancy *Anh* et al. showed that periostin gene expression in the bovine endometrium is triggered by progesterone, while interferon tau seems to lower local PER levels [21]. On the other hand, Hiroi et al. describe that PER-levels are increased during the proliferative phase and decrease in the second half of menstrual cycle [22].

In addition, PER plays an important role in embryo development as it was shown to be present at amniotic membranes and to be higher expressed in neonatal umbilical cord than in adult serum [23–25]. Furthermore, an important pathway during early pregnancy, Wnt-pathway,is targeted by PER [26].

Morelli et al. demonstrated in a case-control-study that PER mRNA and protein levels were lower in decidual and trophoblastic tissues from women with miscarriages in late first trimester compared to women with intact pregnancies, who underwent abortion induction. They concluded therefore that PER represents a marker of a viable pregnancy in the first trimester [18]. However, since this analysis included patients at 12 weeks of gestation when the miscarriage already had occurred and tissue was examined rather than female serum, these results do not allow prognostic conclusions for the outcome of the pregnancy during the first trimester.

As PER has been described as a biomarker in various medical conditions [14, 27, 28] we evaluated if changes in PER serum levels may help identify women at risk for spontaneous abortion in the first trimester.

## Methods

After informed consent, 41 pregnant women in the study who had undergone ovarian hyperstimulation for in vitro fertilization with or without intracytoplasmic sperm injection were included. Inclusion criteria were: age between 18 and 42 years, pregnancy after ovarian hyperstimulation followed by embryo transfer. Women with possible confounding comorbidities (autoimmune diseases, essential hypertonia, diabetes mellitus or the intake of confounding medication) or a history of recurrent miscarriage or implantation failure were excluded.

The study was approved by the Ethical Committee of Heidelberg University (protocol S-243/2015). Maternal serum samples were obtained at the time of pregnancy testing (PT) and the following first ultrasound checkup (US) after approximately 10 days (9.9 ± 1.5 (group 1) days vs. 10.7 ± 2.1 (group 2) days) for the measurement of PER. Pregnancies were adequately developed with normal HCG rise at the time of the first US. Patients with a positive pregnancy test were subsequently divided in two groups: (1) patients with subsequent miscarriage in the first trimester (missed abortion, incomplete spontaneous abortion or complete spontaneous abortion, *n* = 18) later in pregnancy and (2) patients with ongoing pregnancy (*n* = 23).

### ELISA assays

PER levels were analysed using the AdipoGen® Elisa kit for Human Periostin, catalog no. AG-45B-004-KI01, Lot K2321605, Adipogen International, Liestal, Switzerland. Serum samples were obtained and centrifuged for 20 min, 1200 x g, 10 °C. ELISA reagent (Wash Buffer, ELISA buffer, Detection Antibody, HRP labeled Streptavidin and Human Periostin standard) preparations were performed as described by the manufacturer.

Standard curve was prepared as described by the manufacturer, obtaining concentrations of 5000 pg/ml, 2500 pg/ml, 1250 pg/ml, 625 pg/ml, 312 pg/ml, 156 pg/ml, 78 pg/ml and 0 pg/ml). Samples were diluted 1/50 in ELISA buffer, as recommended.

100 μl of different standards and 100 μl of diluted serum were added to each well of the prepared microplate in duplicates. After incubation (2 h at 37 °C), wells were aspirated and washed. 100 μl of diluted Detection Antibody was added to each well, followed by incubation for 1 h at 37 °C. Again, supernatant was aspirated and the coated wells were washed. Afterwards 100 μl of diluted HRP labeled Streptavidin was added. After incubation for 30 min at room temperature, supernatant was aspirated and wells were washed, before 100 μl of TMB substrate solution were overlayed. Color reaction was allowed to develop in the dark for 15 min before 50 μl of stop solution were added.

Optical density of each well was measured using a microplate reader set to 450 nm with wave length correction of 570 nm.

### Statistical analysis

Statistical analysis was performed using the Kolmogorov–Smirnov-test, the Fisher-exact-test and unpaired Student’s t test. A *p*-value ≤ 0.05 was considered to be significant.

## Results

Descriptive data of patients are demonstrated in Table 1. There was no significant difference in age (34.2 ± 4.0 (group 1) years vs. 33.0 ± 6.3 (group 2) years) or body mass index in group 1 and 2 with 25.8 ± 5.1 kg/m^2^ in group 1 vs. 24.4 ± 4.4 kg/m^2^ in group 2. Embryo quality was similar in both groups. We grouped the embryos based on “The Istanbul consensus workshop on embryo assessment” [29] and had a transfer of poor embryos in 3 of 18 patients with miscarriage (16.7%) and in 8 of 23 patients with ongoing pregnancy (34.8%). Fishers exact-test revealed a *p*-value of 0.29 (Table 2). Additionally, no statistically significant difference in day of transfer with 3.8 ± 1.3 in group 1 vs. 4.5 ± 1.0 in group 2 (Table 2) or in time between pregnancy test and ultrasound control with 9.9 ± 1.5 days in group 1 vs. 10.7 ± 2.1 days in group 2 (Table 1) was observed. All values are given in mean ± STD.

**Table 1:** Descriptive data of patients

Nr	Abortion	Ongoing pregnacy	Age	BMI	PT	US	t(US-PT(d))
1	yes	no	38	21.4	4 + 0		
2	yes	no	26	30.1	4 + 3		
3	yes	no	32	23.3	4 + 0	5 + 2	9
4	yes	no	34	21.9	4 + 0		
5	yes	no	39	20.2	4 + 0		
6	yes	no	33	33.1	4 + 3	5 + 5	9
7	yes	no	36	24.9	4 + 2		
8	yes	no	35	22.0	4 + 0		
9	yes	no	41	30.7	4 + 0	5 + 4	11
10	yes	no	32	24.4	4 + 0	5 + 5	12
11	yes	no	36	35.2	4 + 0	5 + 3	10
12	yes	no	35	30.8	4 + 0	5 + 5	12
13	yes	no	34	29.7	4 + 0	5 + 0	7
14	yes	no	31	22.5	4 + 0		
15	yes	no	30	20.0	4 + 0		
16	yes	no	40	23.0	4 + 0	5 + 3	10
17	yes	no	35	32.0	4 + 0	5 + 3	10
18	yes	no	28	19.3	4 + 0	5 + 2	9
19	no	yes	29	23.7	4 + 0		
20	no	yes	34	21.5	4 + 0	5 + 4	11
21	no	yes	31	19.7	4 + 3	5 + 6	10
22	no	yes	33	23.6	4 + 0	5 + 3	10
23	no	yes	34	25.4	4 + 0	5 + 4	11
24	no	yes	37	27.0	4 + 0		
25	no	yes	41	21.5	4 + 0		
26	no	yes	32	25.4	4 + 0	5 + 4	11
27	no	yes	31	29.0	4 + 0		
28	no	yes	35	24.7	4 + 0		
29	no	yes	30	27.3	4 + 0	6 + 5	18
30	no	yes	10	38.2	4 + 1	5 + 4	10
31	no	yes	35	27.1	4 + 0	5 + 3	10
32	no	yes	29	21.0	4 + 0	5 + 2	9
33	no	yes	37	25.7	4 + 0	5 + 3	10
34	no	yes	38	21.3	4 + 0	5 + 3	10
35	no	yes	41	24.1	4 + 0	5 + 3	10
36	no	yes	33	27.0	4 + 0	5 + 4	11
37	no	yes	41	23.2	4 + 0	5 + 4	11
38	no	yes	29	30.1	4 + 0		
39	no	yes	30	19.5	4 + 0	5+4	11
40	no	yes	37	18.6	4 + 0	5 + 2	9
41	no	yes	33	18.3	4 + 0	5 + 3	10
ttest			ns	ns			ns

*PT*, pregnancy testing, *US*, ultrasound control, *ns*, not significant

**Table 2:** Descriptive data of embryos transferred

Nr	Abortion	Ongoing pregnacy	day of embryo transfer	Embryo quality
1	yes	no	day 5	4AA, 4AA
2	yes	no	day 4	11A
3	yes	no	day 4	4B, 5B
4	yes	no	day 5	4AB, 4AA
5	yes	no	day 2	4B
6	yes	no	day 4	Blastocyst 1, Blastocyst 2
7	yes	no	day 5	Blastocyst 1, Blastocyst 2
8	yes	no	day 2	4A, 4A
9	yes	no	day 2	2A
10	yes	no	day 5	8A, 8C
11	yes	no	day 2	8B, 7C
12	yes	no	day 5	Blastocyst 1, Blastocyst 2
13	yes	no	day 4	4BA, 3AA
14	yes	no	day 3	4AA, 4AA
15	yes	no	day 2	9C, 5B, 8C
16	yes	no	day 5	4AA, Blastocyst 2
17	yes	no	day 5	3AB, Blastocyst 2
18	yes	no	day 5	3BB
19	no	yes	day 4	12B
20	no	yes	day 5	4AA hatching
21	no	yes	day 2	2A, 4B
22	no	yes	day 2	5B
23	no	yes	day 5	Blastocyst 2
24	no	yes	day 3	9B
25	no	yes	day 4	Morula
26	no	yes	day 5	4BB, 4BB
27	no	yes	day 5	Blastocyst 1, Blastocyst 2
28	no	yes	day 5	Morula, Blastocyst 1
29	no	yes	day 5	Blastocyst 1
30	no	yes	day 5	3BA
31	no	yes	day 5	4AA
32	no	yes	day 5	Blastocyst 1
33	no	yes	day 4	4AB, 3AB
34	no	yes	day 5	Morula, Blastocyst 1
35	no	yes	day 5	4BA, Blastocst 1
36	no	yes	day 5	Blastocyst, 3BA
37	no	yes	day 5	Blastocyst 1
38	no	yes	day 4	Morula, Morula
39	no	yes	day 5	4AA, 4AA
40	no	yes	day 5	4AB, 3AB
41	no	yes	day 5	3BA, 4AA
ttest			ns	

We obtained samples from both time points in 10 of 18 patients in group 1 (55.6%) and in 17 of 23 patients in group 2 (73.9%), allowing to investigate if there were any temporal or relative changes between pregnancy test and the time of the ultrasound control between the two groups.

Absolute and relative concentrations of PER by Kolmogorov–Smirnov-test revealed a normal distribution pattern.

We did not observe any significant difference in PER levels at the time point of pregnancy testing or the time point of ultrasound control (Table 3).

**Table 3:** Results of Periostin assessment

	Group I (abortion)			Group II (control)	
	n		n		ttest
Pregnancy test (PT)	18	22,067.22 ± 1445.5 pg/ml	23	26,736.52 ± 2562.96 pg/ml	ns
Ultrasound Control (US)	10	21,875.00 ± 1256.55 pg/ml	17	25,855.00 ± 2516.38 pg/ml	ns

All values are given in mean ± SEM

However, by investigating the temporal changes of PER levels within the two groups, we observed that patients with a miscarriage showed increasing levels of PER at the time point of PT compared to US (+3622.5 ± 1768.47 pg/ml) in contrast to patients with an ongoing pregnancy who showed a decrease in PER levels (− 1750.29 ± 1461.51 pg/ml, *p* < 0.05) (Table 4).

**Table 4:** Results of Periostin assessment

	Group I (abortion)			Group II (control)	
	n		n		ttest
Pregnancy test (PT)	10	18,252.50 ± 1320.01 pg/ml	17	27,605.50 ± 3450.28 pg/ml	ns
Ultrasound Control (US)	10	21,875.00 ± 1256.55 pg/ml	17	25,855.00 ± 2516.38 pg/ml	ns
US-PT	10	3622.5 ± 1768.47 pg/ml	17	- 1750.29 ± 1447.38 pg/ml	<0.05
US/PT	10	1.24 ± 0.12	17	0.97 ± 0.06	<0.05

All values are given in mean ± SEM. PT-US represents the absolute temporal change in Periostin levels. PT/US represents the relative temporal change in Periostin levels

Based on this observation, we built the ratio of PER expression at the time point of first ultrasound relative to pregnancy testing. The relative expression of PER was significantly higher in patients with miscarriage compared to control patients with ongoing pregnancy (1.24 ± 0.12 vs. 0.97 ± 0.06, *p* < 0.05).

Ratio of hCG-levels between the 6. week of gestation and the time of pregnancy testing did not differ significantly between the two groups (53.46 ± 54.01 in patients with miscarriage vs. 67.79 ± 54.37 in women with ongoing pregnancy, *p* = 0.52).

## Discussion

This study is the first study, to the best of our knowledge, evaluating systemic levels of PER in the first weeks of pregnancy in order to investigate the differences between women who are going to abort compared to ongoing pregnancy.

As ultrasound cannot determine pregnancy progress, different hormone assessments have been published in order to help predict pregnancy outcome, such as human chorionic gonadotropin (hCG) [30, 31], progesterone [30, 31], kisspeptin [32], activin A [33], activin B [34], follistatin [35], CA-125 [31, 36], pregnancy associated plasma protein A (PAPP-A) [31, 37] or macrophage inhibitory cytokine-1 [38]. However, none of these factors have been established as a biomarker in early pregnancy for miscarriage.

The most appropriate time point to evaluate the risk of miscarriage would be already at the time of pregnancy testing. However, we could not identify any significant changes in PER expression at the time of pregnancy testing. The next possible time point in routine practical use to evaluate a possible screening biomarker for miscarriage is the time point of ultrasound control in the 6th week of gestation. Again, we could not identify significant changes between the two groups. This observation may be due to high interindividual variations and the small sample size, however the intraindividual variation seems to be low, as the sequential values show significant changes over time. Therefore we aimed to investigate if there were any intraindividual alterations in the temporal expression to differentiate between the two groups. The relative expression of PER over time was significantly higher in patients with miscarriage compared to control patients with ongoing pregnancy. The fact that PER is overexpressed in different diseases characterized by inflammation [8] can represent a possible explanation for our observations. The important role of PER in embryo invasion has been described as well as local PER expression at 12 weeks of gestation when miscarriage has already occurred [18, 23]. However, none of these studies evaluated systemically serum levels. Additionally, our study assesses PER levels in an earlier period of gestation.

Intraindividual relative expression, which eliminates the risk of confounding comorbidities, of PER over time represents a new possibly independent tool in risk assessment. However one of the limitations of our study is its small sample size and the fact that not all participations presented themselves in the 6. week of gestation, further minimizing our sample size, therefore our results need further validation in larger study cohorts in order to define a precise threshold at this very early stage in pregnancy to develop a predictive test.

## Conclusion

In conclusion, we have shown for the first time that the relative expression of PER between 4 weeks of gestation and control ultrasound in the 6th week of gestation is significantly altered in asymptomatic women with subsequent miscarriage compared to women with ongoing pregnancy, therefore suggesting systemic PER levels as a potential promising biomarker for pregnancy outcome assessment. The development of an early-screening test to identify patients who are at risk of miscarriage in the actual pregnancy would be useful for several reasons: a miscarriage means an enormous distress for the patient and a predictive test with a negative result could be used to reassure anxious patients [31, 32, 36, 37, 39, 40]. On the other hand, a predictive test with a positive result can warn the patients in a very early stage of pregnancy [39], and will prohibit unnecessary prolongation of the current pregnancy by supplementation of high doses of progesterone, as often done in women after ART.

## Abbreviations

ART: Assisted Reproductive Technology
ICSI: Intracytoplasmic sperm injection
IVF: In vitro fertilization
PER: Periostin
PT: Pregnancy testing
US: Ultrasound checkup
